# The effectiveness of trauma-informed youth justice: a discussion and review

**DOI:** 10.3389/fpsyg.2023.1157695

**Published:** 2023-09-08

**Authors:** Andrew Day, Catia Malvaso, Carolyn Boyd, Katherine Hawkins, Rhiannon Pilkington

**Affiliations:** ^1^School of Social and Political Sciences, University of Melbourne, Parkville, VIC, Australia; ^2^School of Health Science, Swinburne University of Technology, Hawthorn, VIC, Australia; ^3^School of Psychology and the School of Public Health, University of Adelaide, Adelaide, SA, Australia; ^4^School of Psychology, University of Adelaide, Adelaide, SA, Australia; ^5^South Australian Department of Human Services, Adelaide, SA, Australia; ^6^School of Public Health, University of Adelaide, Adelaide, SA, Australia

**Keywords:** youth justice, trauma informed practice, indicators, performance, measurement, outcomes

## Abstract

Youth justice services around the world are under increasing pressure to find new and more effective ways of working with young people. One way forward is to implement a more compassionate approach to service delivery that embraces the idea of ‘trauma-informed practice’. And yet, substantial variation has been observed in how a trauma-informed approach has been defined and understood by practitioners, with idiosyncratic implementation evident across different systems and only limited evidence that this results in reductions in subsequent re-offending. In this paper we argue that the success of efforts to work in more trauma-informed ways cannot be judged using recidivism data alone and that there is a need to identify key indicators of the effectiveness of any trauma- informed approach. We present the case for implementing trauma-informed youth justice and outline key features of the approach. We then present a logic model that articulates key components and identifies short- and longer-term outcomes that can be measured to assess the overall performance of a service. The article concludes with a discussion of the current evidential status of trauma-informed youth justice, identifying areas of current strength and those where further work is needed to develop the evidence base, including the need to demonstrate the hypothesized association between short-term trauma-informed practice outcomes and the longer-term goal of preventing re-offending.

## Introduction

Youth justice services are under increasing pressure to not only develop new, and more effective, ways of working, but also to demonstrate that they are achieving the outcomes that the community expects. Around the western world, a series of reviews, inquiries, and investigations (e.g., Goldson, 2000; Woods and Osho, 2013; Inquiry, 2014; Clancey et al., 2020) have each handed down reports that draw attention to harmful and abusive practice, raising questions about the quality of current services and calling for agencies to articulate a stronger vision to guide their work. In response, youth justice agencies have been encouraged to implement more evidence-informed approaches (e.g., Armytage and Ogloff, 2017), such as differentiated case management based on the Risk Needs Responsivity model (e.g., Brogan et al., 2015), and the adoption of associated assessment tools (e.g., the Youth Level of Service/Case Management Inventory; see Dellar et al., 2023). While this has been shown to improve service outcomes (e.g., Vincent et al., 2021) other approaches, such as the Good Lives Model (presented as more client-centered and strengths-based), have also proven influential (Fortune, 2018). These responses do not, however, always directly address the organizational, administrative, and service contexts in which harmful and abusive practice arises. It is in this context that interest has grown in developing what are referred to as more trauma-informed approaches, with trauma-informed practice (TIP) increasingly identified as a useful way to conceptualize both the activities and the outcomes of a youth justice service (e.g., RCPDCNT, 2017). This, it has been argued, is central to the development of new methods of performance measurement and monitoring (e.g., Mears and Butts, 2008; Myers, 2012) that promote both transparency and accountability. In this paper, we propose a set of quantifiable outcome indicators of a TIP approach and discuss how these can contribute to the overarching goals of reducing risk and increasing community safety.

We begin by describing key features of a trauma-informed approach, before presenting a logic model that identifies how the effectiveness of this approach might best be demonstrated. We conclude with a discussion about the current evidentiary status of this approach. In doing so, we ask some foundational questions about what constitutes ‘success’ in this area and how this might best be measured beyond simple, and often misleading, rate of re-offending indicators. This is important, we suggest, if youth justice agencies are to move beyond rhetoric and aspirational statements to providing services that not only do no harm to young people, but also achieve the longer-term goals of reducing risk and improving community safety.

### What is ‘trauma-informed youth justice’?

The assumptions that sit behind a trauma-informed model of youth justice are different from those that inform the delivery of traditional criminal justice models, in so far as they are not premised on the assumption that punishment and individual deterrence will change behavior. As Griffin et al. (2012) have argued, TIP does not make the distinction between ‘victim’ and ‘perpetrator’ in the same way that most western legal systems do, but rather views ‘risk’ as an understandable reaction to childhood adversity and trauma. The goal of service delivery is thus to provide an environment in which the sequalae of trauma are acknowledged and prioritized such that they can be processed and resolved. Accordingly, the purpose of a trauma-informed youth justice system is not to punish children and young people, but to offer a more compassionate approach that reduces risk by helping young people to feel safer, to better understand their experiences of maltreatment and adversity and, thereby, to recover, heal, and to strengthen their overall wellbeing.

The rationale for adopting a trauma-informed approach is derived, in part, from extensive research showing that a high proportion of young people in receipt of youth justice services will have experienced maltreatment and have a history of contact with the child protection system (e.g., Baidawi and Ball, 2023). A recent systematic review by Malvaso C. G. et al. (2022) reported, for example, consistent evidence from multiple studies conducted across 13 different countries that over 85% of justice-involved young people will report having experienced at least one adverse childhood experience (ACE), with the prevalence of individual ACEs ranging from 12.2% for childhood sexual abuse to 80.4% for parental separation. This is coupled with evidence from the same research team that these experiences are typically associated with clinically significant symptoms of trauma. In their interviews with 184 youth justice clients, the vast majority reported four or more ACEs, with 92% reporting experiencing at least one of these ACEs frequently, adding evidence to support the proposition that these experiences are not isolated events but cumulative experiences that tend to cluster. The majority (nearly nine in 10 young people) scored in the symptomatic range for at least one indicator of trauma symptomatology (such as anxiety, depression, anger, post-traumatic stress, dissociation and sexual concerns), and approximately one in three indicated that they had thoughts about hurting or killing themselves (Malvaso C. et al., 2022; Malvaso C. G. et al., 2022).

TIP is not best conceptualized as a program, but rather as an approach to service delivery that is expected to deliver improved outcomes for staff, children, and young people. There are five foundational principles that underpin the TIP approach: safety; trustworthiness; choice; collaboration; and empowerment (Harris and Fallot, 2001; Substance Abuse and Mental Health Services Administration, 2014) – as well as a further principle, respect for diversity, that is sometimes also applied (see Kezelman, 2014). These connect with four key assumptions that guide practice: *realization* about trauma and how it can affect people and groups; *recognizing* the signs of trauma; having a system which can *respond* to trauma; and *resisting* re-traumatization (Substance Abuse and Mental Health Services Administration [SAMHSA], 2014). Importantly, any attempt at implementation will require activity at all levels of the organization – from those activities targeted at the individual child or young person, to those that support members of staff, through to organizational procedures, policies, and the overall operating philosophy. Branson et al. (2017), for example, have identified the following key domains of activity that define TIP in youth justice and span all levels of the organization: screening and assessment for trauma; services and interventions to address trauma symptomatology; cultural competence; young person and family engagement; workforce development and support; policy and practice that promotes a safe agency environment; and cross-system collaboration. These are useful in so far as they provide a structure that can be used to determine the extent to which TIP has been implemented in practice and, in our view, offer a framework for the potential development of performance indicators relevant to each domain.

However, to the best of our knowledge, a trauma-informed youth justice logic model with clearly defined short- and long-term outcome indicators has yet to be articulated. In this paper we propose that these indicators can be used to measure and to monitor the success of a trauma-informed youth justice service and that will provide more meaningful information than is available when performance is assessed solely by reoffending statistics.

### Efforts to collect evidence to assess the success of trauma-informed youth justice

Beyond demonstrating that the principles of TIP are being adequately operationalized, a major challenge for service providers is to explain how core activities are expected to contribute to the longer-term performance of the agency. Often this connection is either absent or implicit or, at times, contested. For example, it has been suggested that external youth justice stakeholders and mental health providers who work with trauma will typically be focused on symptom reduction and/or improving the wellbeing of young people, while the principal concern of youth justice staff is often on reducing crime and future justice system involvement (Bazemore, 2006). It, therefore, becomes important to show how trauma-informed activities might be expected to lead to more justice-specific outcomes. Ultimately it is this type of evidence that can be used to support arguments for the resourcing required to implement trauma-focused services with integrity.

A starting point for identifying opportunities to collect outcome data is to note that recidivism (or some metric of returns to the criminal justice system) is often used as a key performance indicator of youth justice service effectiveness (Harris et al., 2011; Richards, 2011),^1^ as well as the primary outcome variable in scientific studies investigating the effectiveness and cost-effectiveness of a range of youth justice interventions (e.g., Lipsey, 2009; Lipsey et al., 2010).^2^ The problems with relying on recidivism rates as the only measure of service performance have, however, been well documented. The first of these concerns the validity of this data in relation to the actual occurrence of crime, with Jones et al. (2022) arguing that reconviction rates will inevitably be an under-specification of the construct of any ‘return to offending’. In simple terms, some types of crime are significantly under reported or not always prosecuted, many of those who re-offend will not be convicted, and conversely, some of those who are convicted will have not offended. Moreover, as both Mayson (2019) and Woldgabreal et al. (2020) have pointed out, there are systemic and structural processes that influence who will actually be arrested and convicted (and who will not), with false positives resulting from factors such as levels of surveillance and policing, and/or racism. Jones et al. (2022) also discuss how individuals will vary markedly in terms of what they term ‘conviction evasion skills’, namely their willingness to confess, and/or their access to legal representation. There are also considerable problems with interpreting the significance of any changes in reconviction rates of cohorts of children and young people over time, given that offending behavior is expected to increase over the course of adolescence, before declining into early adulthood (i.e., the ‘age crime curve’, see Shulman et al., 2013). This makes it difficult to determine with any certainty whether reductions in recidivism reflect natural ‘aging out’ processes, or whether they can be attributed to the impact of specific interventions (and this is particularly true when interventions are provided years prior to any decline in re-offending being observed). A second issue concerns inconsistent or unreliable measurement and the use of proxy measures of re-offending. For example, Siennick and Pupo (2022), writing in the US context, show how between-state variation in the operationalization of recidivism impacts on ratings of risk, e.g., the most common proxy event tracked by US states was re-adjudication or reconviction, though only about half of states tracked this and follow-up periods varied from between 6- and 36-months post-supervision.

The main limitation of relying on reconviction statistics though, in our view, is that they tell us very little about the mechanisms through which youth justice services influence future behavior. So, while we do know that custodial sentencing will have only a minimal impact on reducing reoffending in young people (Gavin, 2014) and will often increase risk (McAra and McVie, 2007), recidivism data tell us almost nothing about specific activities or programs that ensure children and young people avoid ongoing contact with the criminal justice system. It is here that it becomes important to articulate how these relate to the actual activities of a youth justice service or how constituent programs and services are expected to combine to produce outcomes. While, at present, there are tools available to audit the extent to which a service delivers TIP (i.e., the integrity of TIP; Fallot and Harris, 2006), these do not consider whether the anticipated outcomes of TIP occur. And this includes both short-term changes and those longer-term outcomes that are currently measured using reoffending statistics.

Our recent umbrella review (Boyd et al., under review) synthesized the findings of a number of recent systematic reviews and meta-analyses summarizing the current evidence base supporting the use of different elements of TIP in youth justice settings. We concluded that, overwhelmingly, the body of current evaluation evidence concerns just one component of TIP – the provision of trauma-specific treatment – with relatively little data available that speaks to the other TIP domains identified by Branson et al. (2017). We argued that the evidence to support the delivery of trauma treatment with justice-involved young people was “inconclusive” (p. xx), although we concluded there are sufficient grounds to offer treatment for young people experiencing trauma as well as to provide specialist training and support for staff. However, we also argued that there is only limited evidence that this type of treatment will result in any behavioral change that is specifically related to re-offending. As Baglivio and Wolff (2021) have also suggested, while specific trauma treatments for justice-involved young people do appear to reduce trauma-related symptoms, their impact on reducing subsequent offending is somewhat limited (see also Kerig and Becker, 2010; Zettler, 2021).

Two key studies have looked specifically at the impact of trauma-specific treatment on recidivism following release (Ford and Hawke, 2012; Smith et al., 2012), but have produced conflicting results. This may, in part, reflect the need to better identify (and measure) the hypothesized mechanisms of change and how these translate into measurable rehabilitative outcomes. For example, the reduction of dynamic risk is often identified as the key mechanism by which a reduction in subsequent offending is expected to occur, despite insufficient evidence that measurable reductions in dynamic risk are reliably associated with rehabilitative success (Day et al., 2022). There were also methodological issues that arise from the way in which recidivism is measured (e.g., only official arrests vs. official arrests plus self- and caregiver-reported delinquent behavior), that again highlights the importance of identifying valid and reliable ways of measuring key outcomes. And so, in this article, we propose that other outcomes and indicators can, and should, be used to demonstrate the success of a trauma-informed youth justice service.

### A program logic for trauma-informed youth justice

The term ‘logic model’ is used to refer to a chain of assumptions that explain how program activities lead, step-by-step, to the desired outcomes (W.K. Kellogg Foundation, 2004; Patton and Patrizi, 2010). The most commonly used logic model is a pipeline, which is a simple, linear representation of common elements (Table 1). Logic models are used to visually represent a plausible and sensible method of how a program works under specific conditions to solve an identified problem (Dwyer and Makin, 1997). This requires all key components of a program or service to be articulated along with their intended short- and long-term effects.

**Table 1:** Logic model domains.

Domain	Description
Resources or inputs	The human, financial, organizational, and community resources a program has available which are directed toward doing the work.
Program activities	What the program does with those resources, including the processes, tools, events, technology, and actions that are an intentional part of program implementation. Interventions are used to bring about the intended program changes or results.
Outputs	Direct products of program activities and may include types, levels, and targets of services to be delivered by the program (i.e., the simplest and most immediate indicators of progress).
Outcomes	Specific changes in program participants’ behavior, knowledge, skills, status, and level of functioning. Medium-term outcomes should be attainable within 1- to 3-years, while longer-term outcomes should be achievable within a 4-to-6-year timeframe.
Impact	Fundamental intended or unintended change occurring in organizations, communities or systems that result from program activities within 7- to 10-years.

Logic models summarize the overall mechanism of change by linking processes to eventual effects and thereby helping evaluators to identify relevant process and outcome evaluation measures. The logic model approach is commonly used in the integration of planning, implementation, evaluation, and reporting of programs (Taylor-Powell and Henert, 2008) and thus seems a good starting point for the efforts to collect evidence to assess the success of trauma-informed youth justice. They also help to articulate the underlying program theory (in terms of plausibility, consistency with evidence, and utility), and a theory of action that explains the activities undertaken and what level of success is necessary for each outcome to produce the expected results. This is important in relation to TIP, which has been described as inconsistently operationalized, with the term adopted to describe a diverse range of service responses (Branson et al., 2017).

There is no single accepted logic model to describe TIP in youth justice, as services and agencies operate in different contexts with different mandates. Generally, however, the success of trauma-informed youth justice is conceptualized in terms of the extent to which the underlying principles of a trauma-informed approach have been operationalized and implemented, including in terms of how this results in changes to the lived experience of justice-involved young people.

**Figure 1:**
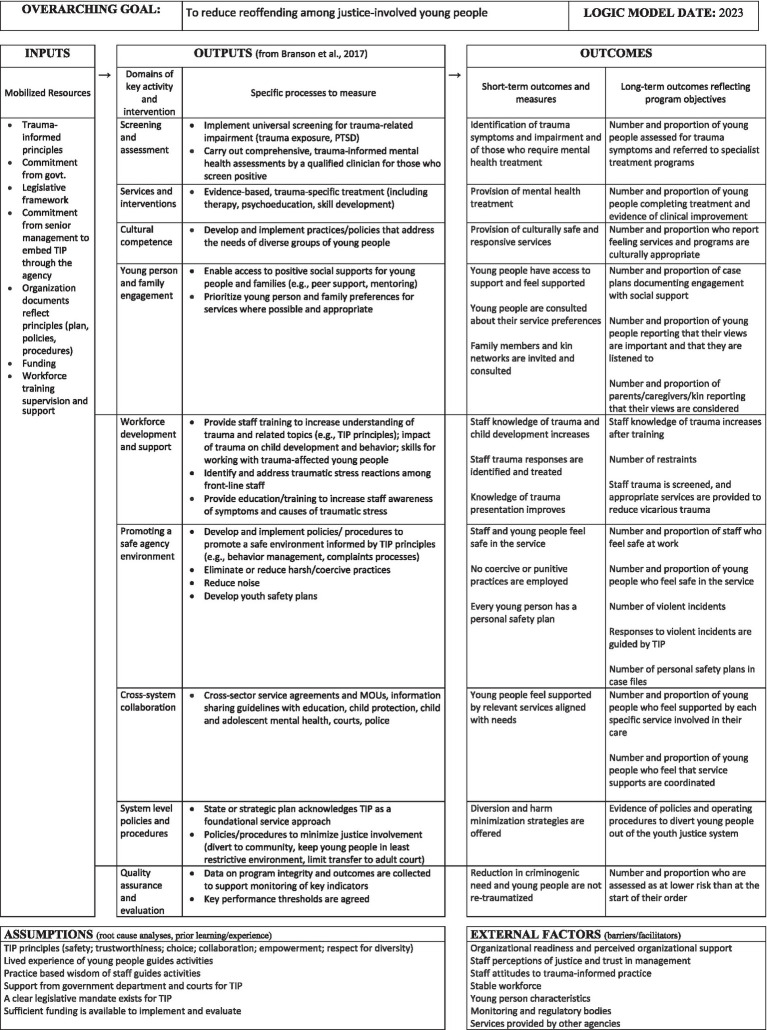
Example of an illustrative logic model for trauma-informed youth justice. Figure adapted from University of Wisconsin Extension Program Development and Evaluation resources: http://www.uwex.edu/ces/pdande/evaluation/evallogicmodel.html.

In effect, these principles represent some of the core assumptions that are made about change. They connect well with the seven key domains of activity described by Branson et al. (2017) in their attempt to describe TIP service delivery, which we have used to develop an illustrative logic model for youth justice TIP that includes expected short- and long-term outcomes (Figure 1). This, we suggest, offers a more nuanced way to understand key interim outcomes that, in theory at least, logically contribute to the longer-term goal of reducing risk and, ultimately, the frequency and severity of re-offending.

The overarching logic of TIP as described in our model is that the longer-term outcomes described in Figure 1 will collectively also contribute to a reduction in re-offending. And, given the methodological problems that are inevitably associated with relying on criminal justice recidivism statistics alone (see above), it makes sense to develop an approach to systematically measuring the extent to which these outcomes are being delivered. This model can serve as the basis of an indicator approach to assessing the performance of a service based on the identification of a specific set of indicators logically associated with TIP core activities.

An indicator provides a common way of measuring and presenting information that reveals whether a specific service standard is being met. It can be based on both quantitative values (e.g., the number of young people who receive a program) on a particular census date or over a period of time, and/or the existence of relevant policy or self-report data about the experience of a service (e.g., feeling safe). A set of indicators will never provide information about all possible aspects of a service but should offer a basic dataset and comparative tool that can then be used to support monitoring and evaluation and to inform policy development. And, if coupled with more qualitative data from those with lived experience of the service, should provide a detailed and accurate picture of overall service performance. For example, the United Nations Office on Drugs and Crime (UNODC, 2006) have developed a manual for the measurement of juvenile justice indicators based on a set of 15 indicators, 11 of which are quantitative and 4 relate to policy. They have been designed to provide an ongoing method of monitoring outcomes but have not been specifically tailored to measuring TIP in youth justice. Hence, a different set of indicators is presented in Figure 1. This depicts how individual indicators of ‘success’ might be organized according to the ‘distance’ between the underlying phenomenon being measured and the direct outcome measure. These are proximate indicators that, theoretically, all contribute to a reduction in re-offending.

Our proposed logic model can also accommodate the influence of how broader conditions influence outcomes by changing relevant social, cultural, or economic conditions. These are important in terms of determining the threshold that must be passed for a particular outcome to be considered to be sufficient for behavioral change to have occurred. Thus, for example, it is not realistic to expect a TIP youth justice service to cause a significant reduction in the overall rate of recidivism when it is responsible for managing a high proportion of short-sentence or unsentenced young people. Even when the long-term objectives of TIP are achieved (e.g., the number of young people being screened for trauma presentations and being referred to treatment), there are external and environmental factors that constrain the likely impact of these services and activities. Rather, the selection of indicators of service performance is based on proximity (the most direct measures of progress are preferred); relevance (those that are most closely associated with re-offending in the community); accuracy (low variation to ensure that an indicator is suitable for understanding cause and effect); and frequency (the more frequent the measurement, the better quality of data). In this way it becomes possible to benchmark change relative to the ‘best’ and ‘worst’ outcomes, allowing progress to be tracked against time, as well as against a ‘frontier’ of best possible outcomes.

For Day et al. (2022), three possible proximate indicators of progress are identified as relevant to rehabilitative success for adults in prison: awareness of dynamic risk; commitment to and optimism about change; and social support for change, whereas in our logic model the indicators have been selected to reflect the different domains of key TIP activity and interventions identified by Branson et al. (2017). These different indicators can be grouped according to four ecologically-framed levels: the young person (and their family); the practitioner; the service/organization; and the broader system. We provide examples of the types of performance indicators that could be applied across these levels to determine the performance of a trauma-informed youth justice service (Table 2). Information on each set of indicators can then be routinely collected to determine not only the baseline performance of a youth justice service but also, importantly, how performance varies over time.

**Table 2:** Example of ecologically-framed, trauma-informed youth justice performance indicators.

Indicator domain	Example performance indicator for a trauma-informed youth justice	Outcome measure	Source of data
Young person	Indicators relevant to understanding whether young people – and their families – receive a trauma-informed program or service	Number and proportion of young people assessed for trauma symptoms Number and proportion referred to specialist treatment programs Number and proportion of young people receiving/completing treatment and showing evidence of clinical improvement Number and proportion of case plans documenting engagement and collaboration with the young person and with their social support network (e.g., parents/caregivers/kin)	Audit of case plans to identify recorded trauma assessment, appropriate referral on to relevant program/service, outcome of the referral and evidence of receipt of treatment, and evidence of engagement with the young person’s family and social support network.
	Indicators relevant to the perceptions of receipt of a trauma-informed program or service	Number and proportion of young people who report feeling safe most of the time Number and proportion of young people reporting that they are listened to, and that their views are valued Number and proportion of parents/caregivers/kin reporting that their views are considered Number and proportion of parents/caregivers/kin reporting that they feel supported	Social climate survey and interview data from young people
Practitioner	Indicators relevant to understanding whether staff are receiving a trauma-informed program or service to support them in their work	Number and proportion of staff provided with appropriate support services	Audit of staff induction, supervision support and performance management records to identify that screening and referral for support services has occurred.
	Indicators relevant to the perceptions of receipt of a trauma-informed program or service	Number and proportion of staff who feel safe at work most of the time Number and proportion of staff who report feeling safe and supported to raise any work concerns Number and proportion of staff reporting that decision-making in services/organizations is transparent	Social climate survey and interview data from staff
Service/ Organization level	Indicators relevant to the understanding of the safety of the service environment	Number of violent incidents Number of incidents requiring the use of restraints Number and proportion of young people with personal safety plans in case files Number and proportion of staff on workers compensation	Audit of incidents within the service Audit of case files
	Indicators relevant to service/organizational policies and procedures	Development and implementation of a trauma-informed youth justice strategy (by service/organization leaders but also including appropriate consultation with service users and providers) Development and implementation of workforce training package, supports and guidance Number and proportion of staff with a development and training plan in place Managers/supervisors report that practitioners complete training and actively participation in supervision/reflective practice Staff knowledge of trauma increases after participation in training Staff competency in applying principles of trauma-informed practice increases over time Number and proportion of staff (practitioners and leadership) reporting that their wellbeing is supported by the organization Evidence of adherence to policies and operating procedures, e.g., if policy goal it to divert traumatized young people from the youth justice system then an indicator would be the number and proportion of young people successfully diverted	Audit of strategic plans, practice frameworks and guidelines, and operating procedures Audit of staff knowledge of/adherence to trauma-informed policies/practice in different scenarios Audit of service level outcomes, e.g., impact of diversion policy and procedures on practice
System	Indicators relevant to minimizing justice system involvement whenever possible and harm minimization for those who become justice-involved	Evidence of partnership between key stakeholders across the system with shared outcomes of diversion and harm minimization Evidence of appropriate resourcing to support cross-sector collaboration	Audit of cross-sector service agreements and MOUs, information sharing guidelines, and common outcomes

## An outcomes focus in youth justice

The aim of this paper was, first, to illustrate how the success of trauma-informed practice in youth justice might be conceptualized and, second, to offer a way to think about evaluating ‘success’ by moving beyond a reliance only on recidivism data to measure the actual performance of a trauma-informed youth justice service. Given the substantial variation in how TIP has been defined and implemented and the fragmented evidence base that currently exists to support its effectiveness, we have suggested there is a need to explicitly articulate how a trauma-informed approach can be expected to lead to better outcomes for both young people and the community. And the next step here is to identify a set of different short- and long-term outcome indicators that can be used to assess impact and effectiveness.

The context for this work relates to questions about the value and impact of youth justice (notably in relation to the large proportion of young people who will return to the criminal justice system), as well as determining how those activities and programs that are provided might be reasonably expected to change behavior. In essence, we are proposing that youth justice agencies should become more child and developmentally focused in their work, with trauma-informed practice (TIP) identified as a new and promising approach. This is not, however, a suggestion to abandon current models of practice such as the Risk Needs Responsivity (RNR) model (see Brogan et al., 2015). In fact, it is quite possible to introduce a trauma informed approach within contemporary case management and rehabilitation services (see Bates-Maves and O’Sullivan, 2017; Levenson and Willis, 2018), by attending closely to the ways in which adversity and maltreatment may be causal in the formation and expression of criminogenic need (Fritzon et al., 2021), and how this changes as children age and develop (van der Put et al., 2012) as well in relation to both general and individual responsivity (Andrews and Bonta, 2010). Rather, our aim is to draw attention to the lack of current evidence that the adoption of TIP in youth justice will actually result in improved performance (in terms of justice system outcomes, including recidivism). In response, we have presented a TIP logic model and illustrative associated indicators that we hope will provide a basis for evaluating how a trauma-informed youth justice system might result in better outcomes for both young people and the community. We do, however, also acknowledge that different assumptions may underpin trauma-informed youth justice. Griffin et al. (2012) have argued, for example, that trauma-informed practice conceptualizes ‘risk’ in terms of the vulnerabilities that arise in response to childhood maltreatment and social and structural inequalities and that this may lead to the identification of areas where the logic of TIP service delivery departs from that of RNR.

Our starting point in this paper was to present an overview of youth justice TIP. This drew on the work of Branson et al. (2017) which systematically identified key domains of TIP activity, but not performance. We then presented a logic model describing different outcomes, along with a set of possible indicators that can be used to routinely report on the services provided, increase transparency and accountability, and track improvement over time. This, we anticipate, will support efforts to answer the key question posed by Branson et al. (2017) about which of their various recommendations lead to meaningful improvement in outcomes for young people, their families, staff, and the broader agency. However, whereas Branson and colleagues advocate for the commissioning of studies to evaluate the comparative effectiveness of specific trauma-informed practices or policies, in this paper we adopt a more holistic perspective by suggesting it is necessary to achieve multiple outcomes if the long-term goal of reducing re-offending is to be achieved. Nonetheless, we do agree that independent evaluation of the effectiveness of each TIP component remains a priority.

The model presented in this paper identifies a range of example performance indicators for a trauma-informed youth justice service, across four different levels, providing examples of what measurement of outcomes might involve for each. These are, of course, simply illustrations of our proposed approach to implementing trauma informed youth justice, as each service setting will need to determine what is possible in the local context. At the level of the young person, for example, we identify options for measuring whether young people – and their families – receive a trauma-informed program or service and their perceptions of those received. This is presented in terms of simple numerical counts (e.g., *number and proportion of young people who report feeling safe most of the time*), with potential sources of data (and opportunities for new data collection) also identified (e.g., audits of case plans, social climate survey). At the level of the practitioner, we recommend collecting data relevant to understanding whether members of staff are receiving services and support to implement trauma-informed practice as well as information about their perceptions of the quality of such services. At the service level, data relevant to safety is important to collect as well as the presence and appropriateness of relevant policy and procedures. And finally, at the systems level, we suggest that data can be collected concerning efforts to minimize justice system involvement and ongoing harm. These indicators, we argue, will broaden our understanding of service performance beyond that of the evaluation of specific programs.

Given the emerging status of TIP in youth justice settings, we recognize the limitations of our logic model and, indeed, see value in non-linear conceptualizations of how program elements inform and interact with one another. Clearly ongoing evaluation efforts will inform, refine, and populate the basic logic model presented here. We have also relied on the work of Branson et al. (2017) to identify the key activities of TIP and it is likely that these will be elaborated as evidence accumulates and practice matures. For example, we would hypothesize that a greater focus on the peer and social elements of the young person’s environment is probably needed, and that specific outcome indicators could be developed to measure this. We are also aware of related work in other areas (e.g., clinical governance and quality assurance in healthcare, e.g., Azami-Aghdash et al., 2015) that might usefully inform the further development of the ideas presented in this paper.

There is, of course, also a substantial criminological literature to draw upon that sits alongside any efforts to implement TIP in youth justice. This includes work on the understanding and measuring the process of desistance from crime which draws attention to the importance of identity change in the desistance journey (Day and Halsey, 2022). This appears particularly relevant to understanding the changes to identity that often occur during the developmental period of adolescence. Nonetheless, articulating the underlying logic of a trauma-informed approach to youth justice remains an important task given that the current evidence base is somewhat patchy and is typically limited to studies that examine the impact of treatment programs as opposed to TIP approaches (see, redacted for blind review). In fact, there have been relatively few reports in the published literature of data relevant to many of the TIP indicators described in our program logic model. This makes it hard to set thresholds against which judgments can be made about what represents ‘good’ performance; it is not currently possible to benchmark performance against other services.

One of the most striking limitations of our work, however, relates to the evidence to support the argument that implementing TIP will result in a reduction in re-offending in young justice-involved people. It is apparent that calls to introduce TIP in youth justice are often rather general and aspirational in nature rather than based on any demonstrable association between the provision of a trauma-informed service and a consequent reduction in risk. Thus, it might still be argued that the most appropriate service delivery model is one that addresses criminogenic need, as these are the intervention targets that can be expected to be most directly associated with future risk (e.g., Lipsey, 2009). We believe that this remains a viable policy option, while noting the problems that have arisen in trying to demonstrate that justice system programs do lead to a reduction in dynamic risk and, importantly, that this also translates into longer-term behavioral change (see Day et al., 2022 for a discussion).

It remains the case that the rationale for introducing a TIP approach in youth justice is based more on dissatisfaction with current service delivery models than it is with empirical evidence that TIP will produce meaningful improvement in reoffending rates. Currently, the strongest evidence for implementing TIP relates to those programs that aim to assist young people to manage symptoms of trauma rather than the broader ways in which TIP is embedded organizationally. At the same time, we would argue that outcomes cannot be reliably assessed through simple recidivism statistics and that the first step to collecting the type of information needed is to collect evidence that short-term outcomes are being achieved and then measuring longer-term TIP outcomes. These data can then, in theory at least, be triangulated with information about changes in criminogenic need and other valued outcomes from a youth justice service (such as supporting a successful transition into adulthood) to inform policy and practice that is more evidence-based. However, we developed this paper because we believe there is a reasonably strong rationale for expecting services and programs that address the sequalae of trauma and prevent re-traumatization to have a positive impact on subsequent offending behavior. For example, data collected by the National Child Traumatic Stress Network shows childhood adversity to be associated with both internalizing and externalizing behavior problems (e.g., Greeson et al., 2014), with problematic alcohol and substance use among justice-involved adolescents conceptualized as coping mechanisms developed in response to previous, or ongoing, experiences of adversity (Kerig et al., 2009). de Ruiter et al. (2022) have further discussed how emotional numbing and feelings of detachment (that often results from trauma) can lead to callousness and a lack of concern for victims. Even so, questions remain about what a TIP approach might look like in practice and the extent to which it can contribute to the goal of reducing recidivism and enhancing community safety. Ultimately, determining the impact of a service on recidivism is an empirical question that can only be answered by high quality evaluation underpinned by the collection and careful analysis of relevant data.

In conclusion, this paper simply offers one way forward to develop the evidence-base for youth justice services that aspire to be more trauma-informed. Our argument is an empirical one: that relevant short- and long-term outcomes need to first be identified and then measured using an agreed set of indicators if we are to conclude that trauma-informed youth justice offers a more promising way forward than current approaches. We do this by presenting a logic model that articulates key components of a TIP approach to youth justice and identifies key short- and long-term outcomes that can be measured to assess how well a service is performing. We recommend that we move beyond a reliance on recidivism statistics by routinely collecting more relevant administrative data, utilizing methods to collect data about the lived experience of staff and young people (such as surveys, consultations), and holding regular reviews of policy. It should also be possible, of course, to use these types of data to consider the influence of contextual and cohort issues on key outcomes, such as for culturally diverse groups or across different ages and genders. And, again we would argue, that this information should be made publicly available and be used to facilitate wider discussion about the continuous improvement of youth justice services. It is only by trialing such an approach that we will develop an evidence base that allows us to ascertain whether trauma-informed youth justice can achieve the goal of preventing re-offending.

## Author contributions

CM and AD conceptualized and wrote the manuscript. CB, KH, and RP provided critical advice and additional material. All authors contributed to the article and approved the submitted version.

## Funding

CM is supported by an Australian Research Council Discovery Early Career Researcher Award (DE200100679). This research was also supported by funding from the South Australian Department of Human Services, Communities and Justice, Research and Service Development Partnership Grant.

## Conflict of interest

The authors declare that the research was conducted in the absence of any commercial or financial relationships that could be construed as a potential conflict of interest.

## Publisher’s note

All claims expressed in this article are solely those of the authors and do not necessarily represent those of their affiliated organizations, or those of the publisher, the editors and the reviewers. Any product that may be evaluated in this article, or claim that may be made by its manufacturer, is not guaranteed or endorsed by the publisher.

## Footnotes

^1^see https://nij.ojp.gov/topics/articles/measuring-recidivism

^2^see https://www.wsipp.wa.gov/BenefitCost?topicId=1
